# Dimethyl 2-(amino­methyl­ene)malonate

**DOI:** 10.1107/S1600536808012762

**Published:** 2008-05-07

**Authors:** Martin Gróf, Jozef Kožíšek, Viktor Milata, Anton Gatial

**Affiliations:** a Institute of Physical Chemistry and Chemical Physics, Faculty of Chemical and Food Technology, Slovak University of Technology, Radlinského 9, SK-812 37 Bratislava, Slovak Republic; b Institute of Organic Chemistry, Catalysis and Petrochemistry, Faculty of Chemical and Food Technology, Slovak University of Technology, Radlinského 9, Bratislava 81237, Slovak Republic

## Abstract

In the title compound, C_6_H_9_NO_4_, which is an example of a push–pull alkene, N—H⋯O inter­actions stabilize the crystal structure.

## Related literature

For related literature, see: Bouzard (1990 ▶); Chemla & Zyss (1987 ▶); Cook (1969 ▶); Freeman (1981 ▶); Nalwa *et al.* (1997 ▶); Shmueli *et al.* (1973 ▶).
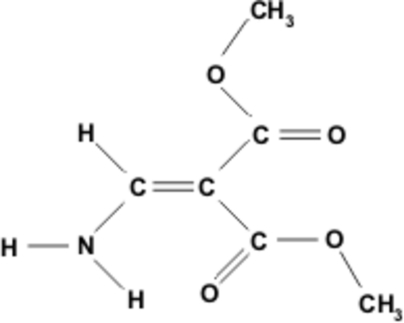


## Experimental

### 

#### Crystal data

C_6_H_9_NO_4_
*M* *_r_* = 159.14
Monoclinic, 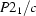

*a* = 9.3410 (19) Å
*b* = 6.9000 (14) Å
*c* = 11.725 (2) Å
β = 97.58 (3)°
*V* = 749.1 (3) Å^3^
*Z* = 4
Mo *K*α radiation
μ = 0.12 mm^−1^
*T* = 100 K
0.43 × 0.27 × 0.06 mm

#### Data collection

Oxford Diffraction GEMINI R diffractometer
Absorption correction: analytical (**CrysAlis RED**; Oxford Diffraction, 2006 ▶) *T*_min_ = 0.892, *T*_max_ = 0.998
14185 measured reflections
1368 independent reflections
841 reflections with *I* > 2σ(*I*)
*R*_int_ = 0.089

#### Refinement

*R*[*F* ^2^ > 2σ(*F* ^2^)] = 0.091
*wR*(*F* ^2^) = 0.261
*S* = 0.99
1368 reflections
102 parameters
H-atom parameters constrained
Δρ_max_ = 0.62 e Å^−3^
Δρ_min_ = −0.38 e Å^−3^

### 

Data collection: *CrysAlis CCD* (Oxford Diffraction, 2006 ▶); cell refinement: *CrysAlis CCD*; data reduction: *CrysAlis RED* (Oxford Diffraction, 2006 ▶); program(s) used to solve structure: *SHELXS97* (Sheldrick, 2008 ▶); program(s) used to refine structure: *SHELXS97* (Sheldrick, 2008 ▶); molecular graphics: *DIAMOND* (Brandenburg, 1998 ▶); software used to prepare material for publication: *enCIFer* (Allen *et al.*, 2004 ▶).

## Supplementary Material

Crystal structure: contains datablocks global, I. DOI: 10.1107/S1600536808012762/bt2703sup1.cif

Structure factors: contains datablocks I. DOI: 10.1107/S1600536808012762/bt2703Isup2.hkl

Additional supplementary materials:  crystallographic information; 3D view; checkCIF report

## Figures and Tables

**Table 1:** Hydrogen-bond geometry (Å, °)

*D*—H⋯*A*	*D*—H	H⋯*A*	*D*⋯*A*	*D*—H⋯*A*
N1—H1*B*⋯O1	0.86	2.02	2.638 (2)	128
N1—H1*A*⋯O2^i^	0.86	2.02	2.848 (2)	161

Symmetry code: (i) 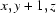.

**Fig. 1.**
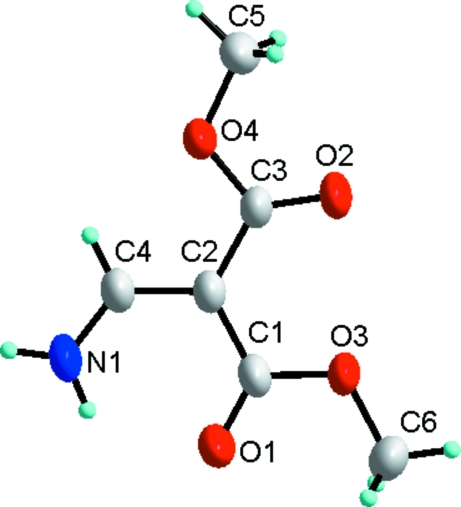
The atom-numbering scheme of aminomethylene-malonic acid dimethyl ester. Displacement ellipsoids are drawn at the 50% probability level.

**Fig. 2.**
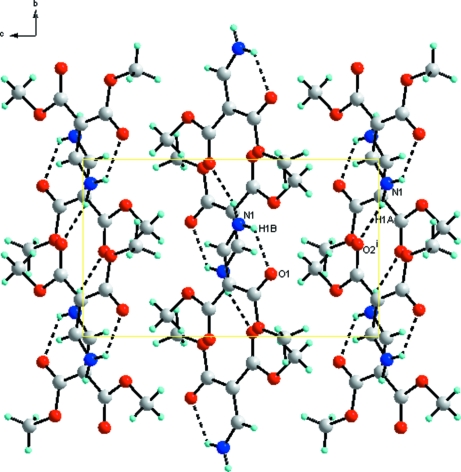
Packing diagram of aminomethylene-malonic acid dimethyl ester. Hydrogen-bond interactions are indicated by dashed lines.

## supplementary crystallographic information

### Comment

The title compound (I), aminomethylene-malonic acid dimethyl ester belongs to the family of so-called push-pull ethylenes. Push-pull ethylenes with the general formula *R*^1^*X*—C*R*^2^=C*R*^3^*R*^4^ are highly reactive organic compounds with electron donor groups at one end and strong electron acceptor groups at the other end of ethylenic C=C double bond. Very often *R*^2^ = H and for *X* = NH or N*R*^1^ and *X* = O as the electron donor groups *R*^1^ can be hydrogen, alkyl, cycloalkyl, aryl or hetero(aryl) groups. On the other side as the electron acceptors *R*^3^, *R*^4^ are groups such as –CN, –COR, –COO*R*, –SO_2_CH_3_, and –NO_2_. Mainly enamines (*X* = NH, N*R*^1^) are frequently used as reactants or intermediates in chemical syntheses of drugs, polymers and dyes (Bouzard *et al.*, 1990, Cook *et al.*, 1969). But also alkoxymethylenes (*X* = O) are often used in organic synthesis (Freeman *et al.*, 1981).

Due to the opposite character of the substituents, the olefinic C=C double bond order is reduced and accompanied by increased bond orders of bonds between the olefinic carbon atoms and their electron donor and electron acceptor groups. This leads to the substantial decrease of the rotational barrier about the C=C double bond and to the increase of an analogues barrier about the adjacent bonds. These changes are connected with the separation of positive and negative charges and electron delocalization within the π-electron system. Such compounds belong to the most developed structures in the search for new compounds with non-linear optics responses (Nalwa *et al.*, 1997, Chemla *et al.*, 1987).

The study of a similar compound, dimethyl-(dimethylaminomethylene)-malonate, has been done  (Shmueli *et al.*, 1973). This study revealed that dimethyl-(dimethylaminomethylene)-malonate exists in solid phase as ZE conformer (*Z* denotes towards to C=C double bond orientation of the carbonyl oxygen in *trans* position; E denotes away from C=C double bond orientation of the carbonyl oxygen in *cis* position). The study of aminomethylene-malonic acid dimethyl ester revealed that this compound exists in solid phase as EZ conformer. The =C—N bond length of 1.301 (4)Å in the title compound is somewhat shorter than in the case of dimethyl-(dimethylaminomethylene)-malonate (1.337 Å). The C=C bond length of 1.385 (5)Å is slightly longer than in the case of dimethyl-(dimethylaminomethylene)-malonate (1.380 Å). The =C—C *trans* and *cis* bond lengths are 1.470 (4)/1.456 (5)Å, respectively in the title compound and 1.442/1.488 Å in dimethyl-(dimethylaminomethylene)-malonate.

### Experimental

To dimethyl 3-methoxymethylenemalonate (1.74 g, 10 mmol) in methanol (10 ml), an aqueous solution of ammonia (12 mmol) was added dropwise (amount according to concentration and density) over a period of 30 min with stirring. The slightly warmed mixture was stirred overnight at room temperature. The reaction mixture was then briefly heated to reflux (ca. 20 min). After ensuring that no starting derivative remained (thin-layer chromatography; Silufol 254, Kavalier Czechoslovakia; eluent chloroform-methanol 10:1 *v*/*v*, detection UV light 254 nm), the reaction mixture was evaporated on a vacuum evaporator and chromatographed on silica gel (eluent dichloromethane-methanol 10:1 *v*/*v*). Obtained product was recrystallized from minimal amount of chloroform and n-hexane mixture in refrigerator.

The solid phase mid-IR vibrational spectrum was recorded with a Nicolet model NEXUS 470 FTIR spectrometer at room temperature. The measurement was performed after mixing the powdered sample with KBr into a pellet.

The mid-IR vibrational frequencies of aminomethylene-malonic acid dimethyl ester are (in cm^-1^): 3460 w; 3358 s; 3296 vw; 3271 vw; 3222 s; 3135 w, sh; 3025 m; 3017 vw, sh; 2993 vw; 2962 m; 2924 vw; 2907 vw; 1683 *vs*; 1658 *vs*; 1637 *vs*; 1576 vw, sh; 1534 m; 1507 s; 1499 s; 1474 vw, sh; 1461 w, sh; 1440 s; 1433 w, sh; 1373 s; 1331 s; 1293 w; 1286 s; 1221 s; 1193 m; 1176 w, sh; 1150 w, sh; 1141 s; 1070 s, b; 1033 w; 982 m; 942 w; 828 w; 822 m; 783 s; 772 w; 750 vw; 715 s, b; 669 w; 649 w, b; 617 m, sh, b; 589 s; 480 s; 440 m.

### Refinement

All H atoms were positioned geometrically and allowed to ride on their corresponding parent atoms at distances of C_methyl_-H = 0.96Å, C_aromatic_-H = 0.93Å and N-H =0.86 Å, with *U*_iso_(H) = 1.2*U*_eq_(C,*N*) or *U*_iso_(H) = 1.5*U*_eq_(C_methyl_). Methyl groups were allowed to rotate but not to tip.

### Crystal data

**Table:**

C_6_H_9_NO_4_	*F*_000_ = 336
*M**_r_* = 159.14	*D*_x_ = 1.411 Mg m^−^^3^
Monoclinic, *P*2_1_/*c*	Mo *K*α radiation λ = 0.71073 Å
Hall symbol: -P 2ybc	Cell parameters from 2135 reflections
*a* = 9.3410 (19) Å	θ = 3.4–29.6º
*b* = 6.9000 (14) Å	µ = 0.12 mm^−^^1^
*c* = 11.725 (2) Å	*T* = 100 K
β = 97.58 (3)º	Block, colorless
*V* = 749.1 (3) Å^3^	0.43 × 0.27 × 0.06 mm
*Z* = 4	

### Data collection

**Table:**

Oxford Diffraction GEMINI R diffractometer	1368 independent reflections
Radiation source: fine-focus sealed tube	841 reflections with *I* > 2σ(*I*)
Monochromator: graphite	*R*_int_ = 0.089
*T* = 100 K	θ_max_ = 25.3º
Rotation method data acquisition using ω and φ scans	θ_min_ = 4.2º
Absorption correction: analytical(*CrysAlis RED*; Oxford Diffraction, 2006)	*h* = −10→11
*T*_min_ = 0.892, *T*_max_ = 0.998	*k* = −8→8
14185 measured reflections	*l* = −14→14

### Refinement

**Table:**

Refinement on *F*^2^	Secondary atom site location: difference Fourier map
Least-squares matrix: full	Hydrogen site location: inferred from neighbouring sites
*R*[*F*^2^ > 2σ(*F*^2^)] = 0.091	H-atom parameters constrained
*wR*(*F*^2^) = 0.261	*w* = 1/[σ^2^(*F*_o_^2^) + (0.1927*P*)^2^] where *P* = (*F*_o_^2^ + 2*F*_c_^2^)/3
*S* = 0.99	(Δ/σ)_max_ = 0.005
1368 reflections	Δρ_max_ = 0.62 e Å^−^^3^
102 parameters	Δρ_min_ = −0.38 e Å^−^^3^
Primary atom site location: structure-invariant direct methods	Extinction correction: none

### Special details

**Table:**

Experimental. face-indexed (*CrysAlis RED*; Oxford Diffraction, 2006)
Geometry. All e.s.d.'s (except the e.s.d. in the dihedral angle between two l.s. planes) are estimated using the full covariance matrix. The cell e.s.d.'s are taken into account individually in the estimation of e.s.d.'s in distances, angles and torsion angles; correlations between e.s.d.'s in cell parameters are only used when they are defined by crystal symmetry. An approximate (isotropic) treatment of cell e.s.d.'s is used for estimating e.s.d.'s involving l.s. planes.
Refinement. Refinement of *F*^2^ against ALL reflections. The weighted *R*-factor *wR* and goodness of fit *S* are based on *F*^2^, conventional *R*-factors *R* are based on *F*, with *F* set to zero for negative *F*^2^. The threshold expression of *F*^2^ > σ(*F*^2^) is used only for calculating *R*-factors(gt) *etc*. and is not relevant to the choice of reflections for refinement. *R*-factors based on *F*^2^ are statistically about twice as large as those based on *F*, and *R*- factors based on ALL data will be even larger.

### Fractional atomic coordinates and isotropic or equivalent isotropic displacement parameters (Å^2^)

**Table:**

	*x*	*y*	*z*	*U*_iso_*/*U*_eq_	
O4	0.9175 (3)	0.2420 (3)	0.85543 (19)	0.0639 (9)	
O2	0.8250 (3)	−0.0207 (4)	0.92664 (19)	0.0657 (9)	
C3	0.8357 (3)	0.1536 (5)	0.9256 (3)	0.0475 (9)	
N1	0.7287 (3)	0.6345 (4)	1.0263 (3)	0.0648 (10)	
H1B	0.6717	0.6127	1.0768	0.078*	
H1A	0.7486	0.7517	1.0089	0.078*	
C2	0.7644 (3)	0.2942 (5)	0.9935 (3)	0.0460 (9)	
C4	0.7847 (4)	0.4902 (5)	0.9761 (3)	0.0511 (9)	
H4A	0.8455	0.5225	0.9222	0.061*	
C5	0.9912 (5)	0.1154 (5)	0.7845 (3)	0.0685 (12)	
H5C	1.0455	0.1916	0.7368	0.082*	
H5B	0.9216	0.0384	0.7368	0.082*	
H5A	1.0555	0.0319	0.8327	0.082*	
O1	0.6120 (3)	0.3524 (4)	1.1347 (2)	0.0773 (10)	
O3	0.6559 (3)	0.0457 (3)	1.0934 (2)	0.0594 (8)	
C1	0.6714 (4)	0.2365 (5)	1.0780 (3)	0.0515 (10)	
C6	0.5661 (4)	−0.0100 (6)	1.1793 (3)	0.0705 (12)	
H6C	0.5600	−0.1487	1.1824	0.085*	
H6B	0.4711	0.0433	1.1596	0.085*	
H6A	0.6075	0.0385	1.2531	0.085*	

### Atomic displacement parameters (Å^2^)

**Table:**

	*U*^11^	*U*^22^	*U*^33^	*U*^12^	*U*^13^	*U*^23^
O4	0.0949 (19)	0.0289 (14)	0.0785 (17)	−0.0010 (12)	0.0514 (14)	−0.0019 (11)
O2	0.094 (2)	0.0233 (15)	0.0884 (18)	−0.0009 (11)	0.0449 (15)	0.0022 (11)
C3	0.065 (2)	0.027 (2)	0.0544 (18)	−0.0036 (14)	0.0236 (16)	0.0017 (13)
N1	0.090 (2)	0.0235 (17)	0.088 (2)	−0.0033 (14)	0.0392 (18)	−0.0022 (14)
C2	0.057 (2)	0.0255 (17)	0.0580 (18)	−0.0039 (14)	0.0172 (16)	0.0018 (14)
C4	0.067 (2)	0.0299 (19)	0.0601 (18)	−0.0043 (15)	0.0210 (17)	0.0008 (15)
C5	0.099 (3)	0.037 (2)	0.079 (2)	0.0036 (19)	0.049 (2)	0.0008 (18)
O1	0.111 (2)	0.0312 (15)	0.104 (2)	−0.0012 (13)	0.0689 (17)	−0.0028 (13)
O3	0.0900 (18)	0.0217 (14)	0.0756 (15)	−0.0054 (10)	0.0445 (13)	0.0026 (10)
C1	0.062 (2)	0.030 (2)	0.066 (2)	−0.0002 (14)	0.0221 (17)	−0.0043 (14)
C6	0.101 (3)	0.039 (2)	0.081 (2)	−0.012 (2)	0.047 (2)	0.0024 (19)

### Geometric parameters (Å, °)

**Table:**

O4—C3	1.342 (4)	C5—H5C	0.9600
O4—C5	1.442 (4)	C5—H5B	0.9600
O2—C3	1.206 (4)	C5—H5A	0.9600
C3—C2	1.470 (4)	O1—C1	1.219 (4)
N1—C4	1.301 (4)	O3—C1	1.340 (4)
N1—H1B	0.8600	O3—C6	1.445 (4)
N1—H1A	0.8600	C6—H6C	0.9600
C2—C4	1.385 (5)	C6—H6B	0.9600
C2—C1	1.456 (5)	C6—H6A	0.9600
C4—H4A	0.9300		
			
C3—O4—C5	115.6 (3)	H5C—C5—H5B	109.5
O2—C3—O4	120.9 (3)	O4—C5—H5A	109.5
O2—C3—C2	127.5 (3)	H5C—C5—H5A	109.5
O4—C3—C2	111.6 (3)	H5B—C5—H5A	109.5
C4—N1—H1B	120.0	C1—O3—C6	116.0 (3)
C4—N1—H1A	120.0	O1—C1—O3	120.4 (3)
H1B—N1—H1A	120.0	O1—C1—C2	123.1 (3)
C4—C2—C1	118.3 (3)	O3—C1—C2	116.5 (3)
C4—C2—C3	119.0 (3)	O3—C6—H6C	109.5
C1—C2—C3	122.8 (3)	O3—C6—H6B	109.5
N1—C4—C2	127.6 (3)	H6C—C6—H6B	109.5
N1—C4—H4A	116.2	O3—C6—H6A	109.5
C2—C4—H4A	116.2	H6C—C6—H6A	109.5
O4—C5—H5C	109.5	H6B—C6—H6A	109.5
O4—C5—H5B	109.5		
			
C5—O4—C3—O2	−0.8 (5)	C3—C2—C4—N1	178.6 (3)
C5—O4—C3—C2	−179.6 (3)	C6—O3—C1—O1	0.1 (5)
O2—C3—C2—C4	−176.8 (3)	C6—O3—C1—C2	178.6 (3)
O4—C3—C2—C4	1.9 (4)	C4—C2—C1—O1	−0.8 (5)
O2—C3—C2—C1	2.5 (5)	C3—C2—C1—O1	180.0 (3)
O4—C3—C2—C1	−178.8 (3)	C4—C2—C1—O3	−179.2 (3)
C1—C2—C4—N1	−0.7 (6)	C3—C2—C1—O3	1.5 (5)

### Hydrogen-bond geometry (Å, °)

**Table:**

*D*—H···*A*	*D*—H	H···*A*	*D*···*A*	*D*—H···*A*
N1—H1B···O1	0.86	2.03	2.638 (2)	128
N1—H1A···O2^i^	0.86	2.02	2.848 (2)	161

Symmetry codes:  (i) *x*, *y*+1, *z*.
